# Echocardiographic left ventricular geometry profiles for prediction of stroke, coronary heart disease and all-cause mortality in the Chinese community: a rural cohort population study

**DOI:** 10.1186/s12872-021-02055-w

**Published:** 2021-05-12

**Authors:** Tan Li, Guangxiao Li, Xiaofan Guo, Zhao Li, Yingxian Sun

**Affiliations:** 1 Department of Cardiovascular Ultrasound, The First Hospital of China Medical University, Shenyang, 110001 People’s Republic of China; 2 Department of Medical Record Management Center, The First Hospital of China Medical University, Shenyang, 110001 People’s Republic of China; 3 Department of Cardiology, The First Hospital of China Medical University, No.155 Nanjing Bei Street, Heping District, Shenyang, 110001 Liaoning Province People’s Republic of China

**Keywords:** Cardiovascular disease, Mortality, Echocardiography, Left ventricular geometry, General population

## Abstract

**Background:**

The utility of echocardiographic left ventricular (LV) geometry in the prediction of stroke/coronary heart disease (CHD) and all-cause mortality is not well characterized. This study aimed to evaluate the overall and sex-specific prognostic value of different geometric patterns on the incidence of stroke/CHD and all-cause mortality in a Chinese population-based cohort.

**Methods:**

We conducted a prospective study in the general population in Northeast China, and a total of 9940 participants aged ≥ 35 years underwent echocardiography for LV geometry and were successfully followed up for incident stroke/CHD and all-cause death. Cox proportional hazards models were utilized to estimate the association of baseline LV geometry with adverse outcomes.

**Results:**

Over a median follow-up of 4.66 years, abnormal LV geometric patterns had increased crude incident rates of stroke/CHD and all-cause mortality compared with normal geometry in overall population and each sex group (all *P* < 0.05). Multivariable Cox analysis reported that LV concentric and eccentric hypertrophy were associated with incident stroke/CHD (concentric hypertrophy: hazard ratio (HR) = 1.39, 95% confidence interval (CI) = 1.04–1.86; eccentric hypertrophy: HR = 1.42, 95% CI = 1.11–1.82) and all-cause mortality (concentric hypertrophy: HR = 1.50, 95% CI = 1.07–2.12; eccentric hypertrophy: HR = 1.58, 95% CI = 1.19–2.10), and LV concentric remodeling was related to stroke/CHD incidence (HR = 1.42, 95% CI = 1.09–1.84) in total population compared to normal geometry after the adjustment for potential confounders. In men, a significant increase was observed from LV eccentric hypertrophy for incident stroke/CHD, whereas in women, LV concentric hypertrophy was associated with elevated incidence of both stroke/CHD and all-cause death, and eccentric hypertrophy was correlated with increased all-cause mortality (all *P* < 0.05).

**Conclusions:**

Our prospective cohort supports that abnormal LV geometry by echocardiography has a prognostic significance for incident stroke/CHD and all-cause mortality, implying that early detection and intervention of LV structural remodeling in rural China are urgently needed to prevent adverse outcomes.

**Supplementary Information:**

The online version contains supplementary material available at 10.1186/s12872-021-02055-w.

## Background

Left ventricular (LV) remodeling can be defined as the process of LV structural change in response to alterations in intrinsic myocardial tissue feature and architecture, or to external stimuli caused by increased pressure or volume overload [1]. Echocardiography that is easily accessible, inexpensive and quick makes possible the recognition of specific LV geometry. In light of LV mass and wall thickness, four echocardiography-derived geometric patterns, including normal geometry, concentric remodeling, concentric hypertrophy and eccentric hypertrophy, are proposed to explain the pathophysiologic basis for cardiac remodeling [2]. The hypertrophic cardiomyopathy and systolic pressure load imposed on LV by hypertension or aortic valve stenosis frequently result in concentric remodeling or hypertrophy, whereas diastolic volume overload imposed on LV in dilated cardiomyopathy, coronary heart disease (CHD), and mitral or aortic regurgitation generally leads to eccentric hypertrophy [3]. Irrespective of etiology, abnormal LV geometry is considered a valuable echocardiographic phenotype that reflects the severity and chronicity of cardiovascular risk factors, suggesting their potential to offer better prognostic information than traditional ones [4–7]. In this regard, the contribution of LV geometry to clinical prognosis has been extensively explored in a variety of cardiovascular disease (CVD) settings.

A number of epidemiological studies demonstrated that LV geometric changes predisposed individuals to increased risk of unfavorable CVD outcomes, including heart failure, cardiac arrhythmias and cerebrovascular disease [8–11]. Conversely, Tovillas-Moran et al. found that the incidence of major adverse cardiovascular events (MACEs) was similar in the four geometric pattern groups, and there was no significant evidence for an association between LV geometry and MACEs in hypertensive patients [12]. It should also be noted that, within subtypes of LV geometry, there is disagreement between studies as to which pattern is the most predictive. Although most population studies have consistently linked LV concentric hypertrophy with poor outcomes [4, 13–15], the reports regarding the adverse impact of LV concentric remodeling and eccentric hypertrophy on prognosis are conflicting [16–18]. Until now, most researches have been restricted to European or American populations and often are quite limited in sample size. However, there is a paucity of data on the possible link between abnormal LV geometric patterns and incident adverse outcomes in Asians. Additionally, the prognostic implications of echocardiographic LV geometry in predicting both cardiovascular events and all-cause mortality are incompletely understood, especially in the general population. And few studies have investigated sex-related influence of LV geometry on the prediction of clinical poor outcomes.

Hence, the purpose of this large-scale epidemiological follow-up study was to assess the overall and sex-specific prognostic significance of different LV geometric patterns by echocardiographic measures for incident stroke/CHD and all-cause mortality in a general Chinese population.

## Methods

### Study population

The Northeast China Rural Cardiovascular Health Study (NCRCHS) is a community-based prospective cohort study carried out in rural areas of Northeast China. The design and inclusion criteria of the study have been described previously [19, 20]. In brief, we recruited a total of 11,956 participants aged ≥ 35 years from Liaoning province between 2012 and 2013 with a multi-stage, randomly stratified and cluster-sampling scheme. At baseline, we excluded subjects who refused echocardiography (n = 341) or had incomplete echocardiographic data (n = 121). Therefore, 11,494 participants were invited to attend follow-up visits in 2015 and 2017. As a result, 10,272 participants consented and were eligible for the follow-up study. A total of 9940 participants (96.8%) completed at least one follow-up visit and were available for analysis. The study was approved by the Ethics Committee of China Medical University (Shenyang, China) and was according to the Declaration of Helsinki. All methods were performed in accordance with the relevant guidelines and regulations. Written informed consent was obtained from each participant. The decease patients are not involved in the study.

### Data collection

Data collection and measurements have been described in our previous studies [20–22]. At baseline, detail information was obtained through the interview with a standardized questionnaire including demographic characteristics, lifestyle factors, disease and medical history. Smoking and drinking status were defined as current use. Weight and height were measured with participants wearing lightweight clothing and no shoes. Body surface area (BSA) was calculated as [0.0061 × height (cm) + 0.0128 × weight (kg)-0.1529]. Body mass index (BMI) was calculated as weight (kg)/height^2^ (m^2^). History of CVD was defined as having a history of CHD, atrial fibrillation (AF), heart failure (HF), stroke or heart valve disease according to subjects’ self-reports. Blood pressure was assessed three times with participants seated after at least five minutes of rest using a standardized automatic electronic sphygmomanometer (HEM-907; Omron, Tokyo, Japan). Hypertension was defined as systolic blood pressure (SBP) ≥ 140 mmHg and/or diastolic blood pressure (DBP) ≥ 90 mmHg, and/or use of antihypertensive medications [23]. Fasting blood samples were collected in the morning from participants who had fasted at least twelve hours. Total cholesterol (TC), triglyceride (TG), low-density lipoprotein cholesterol (LDL-C), high-density lipoprotein cholesterol (HDL-C), fasting plasma glucose (FPG) and other routine blood biochemical indexes were analyzed enzymatically. Diabetes was defined as FPG ≥ 7 mmol/L (126 mg/dl) and/or being on medication for diabetes. Dyslipidemia was defined as serum TC ≥ 6.21 mmol/L (240 mg/dL), or TG ≥ 2.26 mmol/L (200 mg/dL), or LDL-C ≥ 4.16 mmol/L (160 mg/dL), or HDL-C < 1.03 mmol/L (40 mg/dL) and/or under taking hypolipidemic drugs [24].

The method of echocardiography has been described previously [21, 22]. Echocardiographic examination was performed with a commercially available Doppler echocardiograph (Vivid, GE Healthcare, United States) with a 3.0-MHz transducer, including M-mode, 2-dimensional, spectral and color Doppler. Echocardiogram analyses and readings were conducted by three doctors specialized in echocardiography. Under the guideline of the American Society of Echocardiography [25], the parasternal long-axis view was measured to record antero-posterior left atrial diameter (LAD), interventricular septal thickness (IVSd), LV end-diastolic internal dimension (LVIDd), LV end-systolic internal dimension (LVIDs), and posterior wall thickness (PWTd). The LV end-diastolic volume (LVEDV) and LV end-systolic volume (LVESV) were estimated by Teichholz equations: LVEDV (ml) = LVIDd^3^ × 7.0/(2.4 + LVIDd), LVESV (ml) = LVIDs^3^ × 7.0/(2.4 + LVIDs). LV ejection fraction (LVEF) was calculated as [(LVEDV-LVESV)/LVEDV] × 100% and fractional shortening (FS) was determined with [(LVIDd-LVIDs)/LVIDd] × 100%. We applied pulsed-wave Doppler to record the early diastolic peak flow (E) and atrial peak flow (A) of mitral valve in the apical four-chamber view.

Left ventricular mass (LVM) was calculated by the formula: LVM = 0.8 × [1.04{(IVSTd + PWTd + LVIDd)^3^–LVIDd^3^}] + 0.6 g. LVM was divided by BSA to acquire LVM index (LVMI). The relative wall thickness (RWT) was determined with (IVSTd + PWTd)/LVIDd. Ten participants were randomly selected to determine the reproducibility for RWT and LVMI measurements, and the intra-class correlation coefficients for intra- and inter-observer reproducibility for RWT and LVMI were all greater than 0.87 (all *P* < 0.05). LV geometry was classified into four patterns using LVMI and RWT values. They were normal geometry when LVMI ≤ 115 g/m^2^ for men or ≤ 95 g/m^2^ for women and RWT ≤ 0.42; concentric remodeling when LVMI ≤ 115 g/m^2^ for men or ≤ 95 g/m^2^ for women and RWT > 0.42; concentric hypertrophy when LVMI > 115 g/m^2^ for men or > 95 g/m^2^ for women and RWT > 0.42; eccentric hypertrophy when LVMI > 115 g/m^2^ for men or > 95 g/m^2^ for women and RWT ≤ 0.42 [25, 26].

### Definition of clinical outcomes

The median follow-up time was 4.66 (4.36–4.93) years. The number of effective follow-up cases was 9940, and follow-up rate was 96.8%. The outcomes of the present study included stroke/CHD incidence and all-cause mortality. Incident stroke/CHD was defined as new onset stroke or CHD during the follow-up period. Stroke was defined according to the WHO Multinational Monitoring of Trends and Determinants in Cardiovascular Disease (MONICA) criteria as rapidly developing signs of focal or global disturbance of cerebral function, lasting more than 24 h (unless interrupted by surgery or death) with no apparent non-vascular causes [27]. CHD was defined as a diagnosis of hospitalized angina, hospitalized myocardial infarction, any revascularization procedure or CHD-related death [28]. For all participants reporting possible diagnoses or death, all available clinical information was collected including medical records and death certificates. All materials were independently reviewed and adjudicated by the end-point assessment committee. The end-point assessment committee was composed of renowned experts in cardiology, neurology and epidemiology in China, whose responsibility was to judge the event outcomes fairly and accurately.

### Statistical analysis

All the statistical analyses were performed using SPSS version 23.0 software. Continuous variables were reported as mean values ± standard deviations, and categorical variables were represented as numbers and percentages. As appropriate, differences among groups were evaluated with ANOVA or χ2-test. Kaplan–Meier estimates were adopted to calculate the cumulative incidence of stroke/CHD and all-cause mortality for each group, and log-rank test was used to compare the differences in estimates. Multivariable Cox proportional hazards models were utilized to calculate hazard ratios (HRs) with 95% confidence intervals (CIs) for the associations between abnormal LV geometric patterns and stroke/CHD and all-cause mortality. The models were adjusted for baseline age, sex, BMI, smoking, drinking, heart rate, history of CVD, SBP, DBP, TC, TG, LDL-C, HDL-C, FPG, medication for hypertension and diabetes, LAD, LVEF and E/A as appropriate. All tests were two-tailed and *P* < 0.05 indicated statistical significance.

## Results

### Baseline clinical and echocardiographic characteristics

There were 7939 (79.9%) subjects with normal geometry, 759 (7.6%) subjects with concentric remodeling, 426 (4.3%) subjects with concentric hypertrophy and 816 (8.2%) ones with eccentric hypertrophy. The distribution of baseline clinical and echocardiographic features was significantly different among four LV geometry groups (all *P*_*trend*_ < 0.05), as shown in Tables 1 and 2. The clinical and echocardiographic characteristics of the included participants are presented in Additional file 1: Supplemental Table 1.

**Table 1:** Baseline clinical characteristics of the study population

Variables	Normal geometry (n = 7939)	Concentric remodeling (n = 759)	Concentric hypertrophy (n = 426)	Eccentric hypertrophy (n = 816)	*P*_*trend*_
Age, years	52.49 ± 10.07	57.99 ± 10.95	60.79 ± 9.90	59.22 ± 10.05	< 0.001
Male, n (%)	3817 (48.1)	329 (43.3)	175 (41.1)	269 (33.0)	< 0.001
BSA, m^2^	1.66 ± 0.17	1.61 ± 0.19	1.64 ± 0.19	1.60 ± 0.18	< 0.001
BMI, kg/m^2^	24.71 ± 3.62	24.80 ± 3.91	25.86 ± 4.04	25.36 ± 3.90	< 0.001
Smoking, n (%)	2871 (36.2)	259 (34.1)	147 (34.5)	243 (29.8)	< 0.01
Drinking, n (%)	1887 (23.8)	159 (20.9)	90 (21.1)	115 (14.1)	< 0.001
Heart rate, bpm	77.90 ± 12.94	82.02 ± 14.64	80.65 ± 14.59	77.91 ± 13.34	< 0.001
SBP, mmHg	138.50 ± 20.94	150.18 ± 25.62	170.11 ± 26.50	154.55 ± 25.74	< 0.001
DBP, mmHg	80.93 ± 10.87	85.37 ± 12.95	92.61 ± 14.63	85.26 ± 13.34	< 0.001
Hypertension, n (%)	3502 (44.1)	504 (66.4)	395 (92.7)	599 (73.4)	< 0.001
Medication for hypertension, n (%)	847 (10.7)	195 (25.7)	202 (47.4)	227 (27.8)	< 0.001
History of CVD, n (%)	492 (6.2)	99 (13.0)	89 (20.9)	118 (14.5)	< 0.001
TC, mmol/L	5.20 ± 1.06	5.43 ± 1.14	5.65 ± 1.27	5.41 ± 1.15	< 0.001
TG, mmol/L	1.56 ± 1.46	1.74 ± 1.30	1.89 ± 1.41	1.66 ± 1.34	< 0.001
LDL-C, mmol/L	2.91 ± 0.81	3.11 ± 0.91	3.27 ± 0.92	3.04 ± 0.88	< 0.001
HDL-C, mmol/L	1.42 ± 0.39	1.45 ± 0.43	1.39 ± 0.38	1.39 ± 0.35	< 0.01
Dyslipidemia, n (%)	2740 (34.5)	338 (44.5)	201 (47.2)	326 (40.0)	< 0.001
Estimated GFR, mL/min/1.73 m^2^	94.82 ± 15.02	89.48 ± 15.24	85.58 ± 18.50	87.68 ± 15.84	< 0.001
FPG, mmol/L	5.82 ± 1.48	6.23 ± 2.34	6.24 ± 1.79	6.11 ± 1.85	< 0.001
Diabetes, n (%)	712 (9.0)	113 (14.9)	91 (21.4)	131 (16.1)	< 0.001
Medication for diabetes, n (%)	245 (3.1)	48 (6.3)	24 (5.6)	52 (6.4)	< 0.001

*BSA* body surface area, *BMI* body mass index, *SBP* systolic blood pressure, *DBP* diastolic blood pressure, *CVD* cardiovascular disease, *TC* total cholesterol, *TG* triglyceride, *LDL-C* low-density lipoprotein cholesterol, *HDL-C* high-density lipoprotein cholesterol, *GFR* glomerular filtration rate, *FPG* fasting plasma glucose

**Table 2:** Echocardiographic parameters of the study population

Variables	Normal geometry (n = 7939)	Concentric remodeling (n = 759)	Concentric hypertrophy (n = 426)	Eccentric hypertrophy (n = 816)	*P* value
LAD, cm	3.34 ± 0.37	3.30 ± 0.44	3.65 ± 0.46	3.64 ± 0.51	< 0.001
IVSd, cm	0.84 ± 0.08	0.98 ± 0.10	1.14 ± 0.12	0.99 ± 0.10	< 0.001
LVIDd, cm	4.71 ± 0.37	4.27 ± 0.34	4.80 ± 0.40	5.19 ± 0.46	< 0.001
LVIDs, cm	3.11 ± 0.40	2.87 ± 0.39	3.21 ± 0.44	3.42 ± 0.51	< 0.001
PWTd, cm	0.82 ± 0.08	0.94 ± 0.09	1.09 ± 0.10	0.94 ± 0.09	< 0.001
LVM, g	130.40 ± 27.53	135.46 ± 32.16	199.50 ± 44.80	186.57 ± 42.54	< 0.001
LVMI, g/m^2^	78.58 ± 13.26	83.86 ± 19.33	121.35 ± 19.78	115.97 ± 20.35	< 0.001
RWT	0.35 ± 0.03	0.45 ± 0.03	0.46 ± 0.04	0.37 ± 0.03	< 0.001
LVEDV, ml	103.65 ± 19.09	82.44 ± 15.56	108.57 ± 21.36	130.06 ± 29.31	< 0.001
LVESV, ml	39.36 ± 12.31	32.47 ± 11.00	42.55 ± 14.10	49.81 ± 19.78	< 0.001
SV, ml	64.30 ± 15.94	49.96 ± 13.30	66.02 ± 17.11	80.26 ± 21.30	< 0.001
LVEF, %	61.96 ± 9.87	60.58 ± 11.01	60.84 ± 10.25	61.94 ± 10.14	< 0.01
FS, %	33.82 ± 7.13	32.66 ± 7.62	33.07 ± 7.20	34.08 ± 7.35	< 0.001
E wave, cm/s	74.56 ± 19.55	67.38 ± 18.20	63.88 ± 18.05	70.25 ± 22.83	< 0.001
A wave, cm/s	74.62 ± 17.21	81.55 ± 17.78	87.35 ± 20.40	83.46 ± 19.54	< 0.001
E/A	1.05 ± 0.37	0.87 ± 0.33	0.77 ± 0.33	0.88 ± 0.36	< 0.001

*LAD* left atrial diameter, *IVSd* interventricular septal thickness, *LVIDd* left ventricular end-diastolic internal dimension, *LVIDs* left ventricular end-systolic internal dimension, *PWTd* posterior wall thickness, *LVM* left ventricular mass, *LVMI* left ventricular mass index, *RWT* relative wall thickness, *LVEDV* left ventricular end-diastolic volume, *LVESV* left ventricular end-systolic volume, *SV* systolic volume, *LVEF* left ventricular ejection fraction, *FS* fractional shortening, *E* early diastolic peak flow, *A* atrial peak flow

### Incidences of stroke/CHD and all-cause mortality

Over a median follow-up of 4.66 years, a total of 568 stroke/CHD events and 411 all-cause deaths occurred in the studied population. LV concentric remodeling, concentric hypertrophy and eccentric hypertrophy had significantly elevated crude incident rates of stroke/CHD and all-cause mortality in comparison with normal geometry in the overall and stratified analyses (all *P* < 0.05) (Table 3).

**Table 3:** Incidence of stroke/CHD and all-cause mortality at follow-up

Outcomes	Normal geometry (n = 7939)		Concentric remodeling (n = 759)			Concentric hypertrophy (n = 426)			Eccentric hypertrophy (n = 816)		
	n (%)	Rate per 1000 person-years (95% CI)	n (%)	Rate per 1000 person-years (95% CI)	*P* value	n (%)	Rate per 1000 person-years (95% CI)	*P* value	n (%)	Rate per 1000 person-years (95% CI)	*P* value
Overall											
Stroke/CHD	336 (4.2)	9.79 (8.74, 10.84)	73 (9.6)	23.25 (17.92, 28.59)	< 0.001	67 (15.7)	39.33 (29.92, 48.75)	< 0.001	92 (11.3)	27.75 (22.08, 33.42)	< 0.001
All-cause mortality	239 (3.0)	6.85 (5.98, 7.71)	49 (6.5)	14.99 (10.79, 19.19)	< 0.001	47 (11.0)	26.08 (18.57, 33.44)	< 0.001	76 (9.3)	22.06 (17.10, 27.02)	< 0.001
Male											
Stroke/CHD	183 (4.8)	11.08 (9.47, 12.68)	40 (12.2)	29.91 (20.64, 39.18)	< 0.001	31 (17.7)	44.80 (29.03, 60.58)	< 0.001	41 (15.2)	36.97 (25.65, 48.28)	< 0.001
All-cause mortality	129 (3.4)	7.64 (6.32, 8.96)	29 (8.8)	20.70 (13.16, 28.23)	< 0.001	15 (8.6)	19.96 (9.86, 30.06)	< 0.001	29 (10.8)	24.94 (15.86, 34.02)	< 0.001
Female											
Stroke/CHD	153 (3.7)	8.60 (7.24, 9.96)	33 (7.7)	18.31 (12.07, 24.56)	< 0.001	36 (14.3)	35.59 (23.97, 47.22)	< 0.001	51 (9.3)	23.11 (16.77, 29.45)	< 0.001
All-cause mortality	110 (2.7)	6.10 (4.96, 7.24)	20 (4.7)	10.71 (6.02, 15.41)	0.02	32 (12.7)	30.31 (19.81, 40.81)	< 0.001	47 (8.6)	20.60 (14.71, 26.48)	< 0.001

*P* value for the comparison with normal geometry

### Role of abnormal LV geometry in predicting adverse outcomes at follow-up

**Fig. 1:**
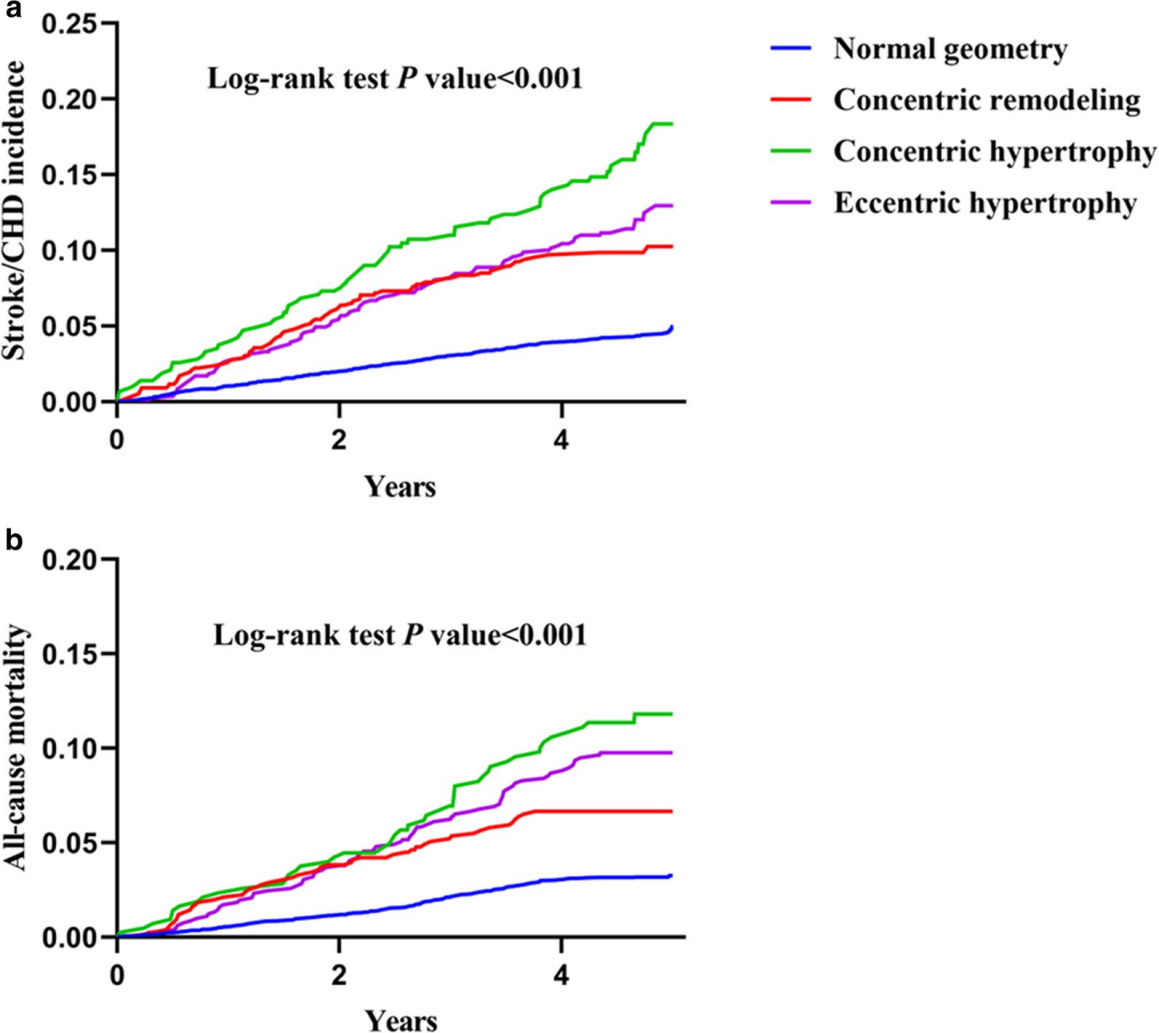
Unadjusted Kaplan–Meier curves for incident stroke/CHD (**a**) and all-cause mortality (**b**) in the overall population

**Fig. 2:**
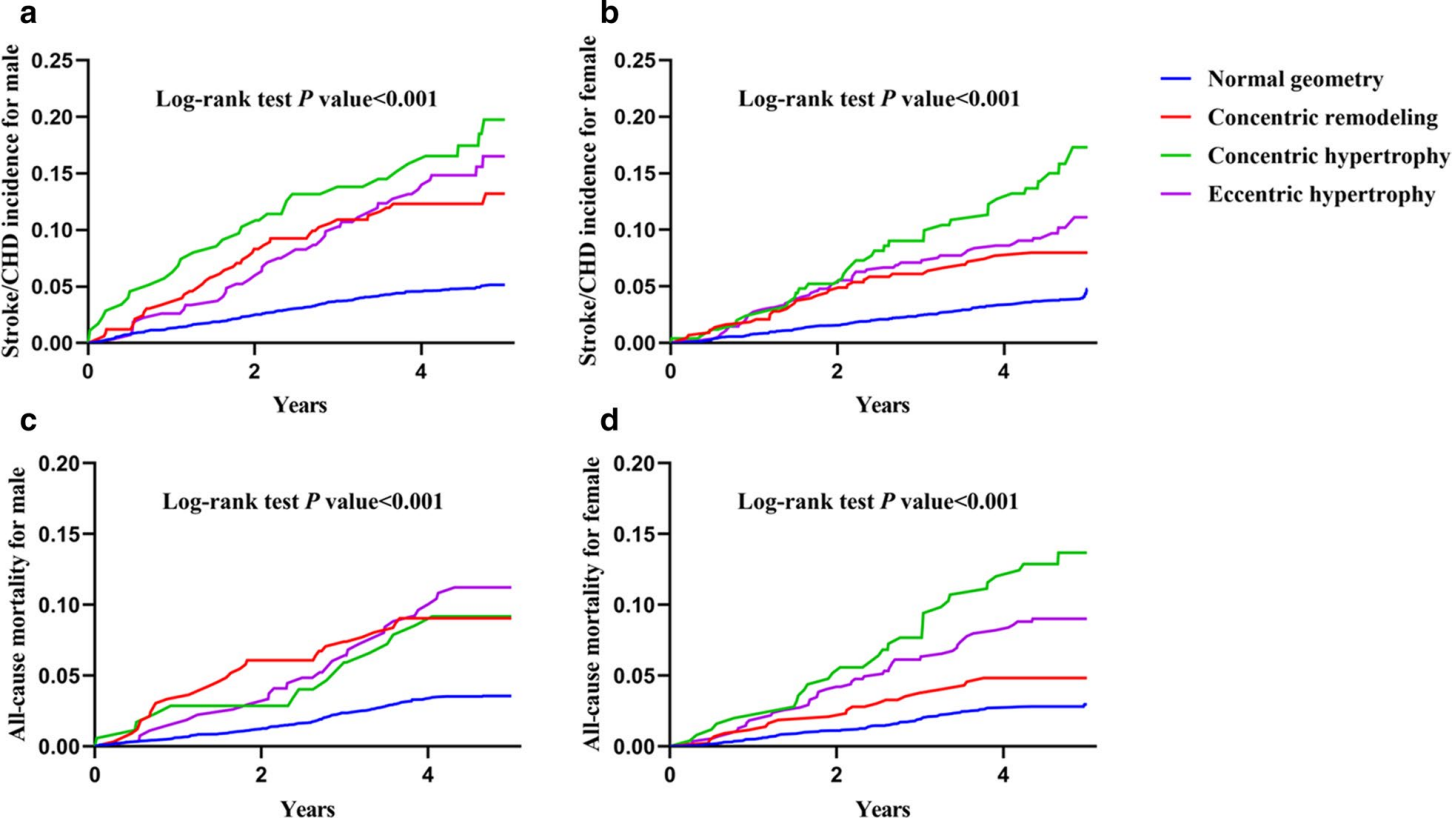
Unadjusted Kaplan–Meier curves for stroke/CHD incidence in male (**a**) and female (**b**), and all-cause mortality in male (**c**) and female (**d**)

According to Kaplan–Meier survival estimates, higher cumulative incidences of stroke/CHD and all-cause mortality were observed among participants with abnormal LV geometry when compared to those with normal geometry in total population (Fig. 1) or each sex group (Fig. 2) (all *P* < 0.001).

Table 4 presents the associations between abnormal LV geometry and adverse outcomes using multivariable-adjusted Cox proportional hazards models. In the overall population, LV concentric remodeling, concentric hypertrophy and eccentric hypertrophy were separately associated with stroke/CHD incidence (concentric remodeling: HR = 1.42, 95% CI = 1.09–1.84; concentric hypertrophy: HR = 1.39, 95% CI = 1.04–1.86; eccentric hypertrophy: HR = 1.42, 95% CI = 1.11–1.82), while concentric hypertrophy and eccentric hypertrophy were correlated with all-cause mortality (concentric hypertrophy: HR = 1.50, 95% CI = 1.07–2.12; eccentric hypertrophy: HR = 1.58, 95% CI = 1.19–2.10) after adjustment for conventional risk factors. In subgroup analyses stratified by sex, LV eccentric hypertrophy was independently related to stroke/CHD incidence in male subgroup (HR = 1.55, 95% CI = 1.08–2.24), whereas in female subgroup, LV concentric hypertrophy had a significant association with incident stroke/CHD (HR = 1.58, 95% CI = 1.06–2.36) and all-cause mortality (HR = 1.99, 95% CI = 1.29–3.08), and eccentric hypertrophy was associated with the risk of all-cause mortality (HR = 1.68, 95% CI = 1.16–2.44).

**Table 4:** Abnormal LV geometry for the prediction of stroke/CHD and all-cause mortality at follow-up

	Stroke/CHD			All-cause mortality		
	Cases/Noncases	Incidence HR (95% CI)	*P* value	Cases/Noncases	Incidence HR (95% CI)	*P* value
Overall						
Normal geometry	336/7603	Reference	–	239/7700	Reference	–
Concentric remodeling	73/686	1.42 (1.09, 1.84)	0.01	49/710	1.23 (0.89, 1.69)	0.21
Concentric hypertrophy	67/359	1.39 (1.04, 1.86)	0.03	47/379	1.50 (1.07, 2.12)	0.02
Eccentric hypertrophy	92/724	1.42 (1.11, 1.82)	< 0.01	76/740	1.58 (1.19, 2.10)	< 0.01
Male						
Normal geometry	183/3634	Reference	–	129/3688	Reference	–
Concentric remodeling	40/289	1.39 (0.98, 1.99)	0.07	29/300	1.50 (0.99, 2.29)	0.06
Concentric hypertrophy	31/144	1.27 (0.83, 1.96)	0.27	15/160	1.01 (0.56, 1.81)	0.98
Eccentric hypertrophy	41/228	1.55 (1.08, 2.24)	0.02	29/240	1.55 (1.00, 2.41)	0.05
Female						
Normal geometry	153/3969	Reference	–	110/4012	Reference	–
Concentric remodeling	33/397	1.36 (0.92, 2.01)	0.12	20/410	0.94 (0.57, 1.55)	0.81
Concentric hypertrophy	36/215	1.58 (1.06, 2.36)	0.03	32/219	1.99 (1.29, 3.08)	< 0.01
Eccentric hypertrophy	51/496	1.27 (0.90, 1.79)	0.18	47/500	1.68 (1.16, 2.44)	< 0.01

Adjusted for age, sex, BMI, smoking, drinking, heart rate, history of CVD, SBP, DBP, TC, TG, LDL-C, HDL-C, FPG, medication for hypertension and diabetes, LAD, LVEF and E/A as appropriate

## Discussion

To the best of our knowledge, this is the first study to investigate the predictive influence of LV geometric patterns on the risk of adverse outcomes in a general Chinese population of nearly ten thousand with a median follow-up of 4.66 years. The major finding of current cohort is that abnormal echocardiographic LV geometry may be an independent and incremental prognostic factor for stroke/CHD and all-cause mortality, and there are some sex differences in the relation between LV geometric patterns and adverse outcomes. Our results may help augment the understanding of LV remodeling-associated stroke/CHD risk and sudden death in clinical practice.

Given that the plasticity of LV remodeling is influenced by differences in adverse stimuli and exposure duration to risk factors, and involves structural, metabolic and functional maladaptive alterations, it is biologically plausible that abnormal LV geometry may add prognostic information for cardiovascular risk [29]. Although LV geometric abnormality has been reported to have a notable prognostic importance and be associated with unfavorable cardiovascular outcomes [4, 8, 17], the contribution of different geometric subtypes to CVD and all-cause death risk is debated [30, 31]. In most population-based prospective studies, LV concentric hypertrophy has been confirmed to carry the greatest risk of future cardiovascular morbid events or all-cause mortality in patients with hypertension, myocardial infarction, AF or ischemic stroke [2, 32–34]. Our cohort demonstrated that LV concentric and eccentric hypertrophy were independent risk factors for both stroke/CHD and all-cause death incidence, while LV concentric remodeling was only associated with the risk of incident stroke/CHD in total population. There are several explanations that may underlie our findings. First, hemodynamics, neurohumoral activation, oxidative stress, coronary microcirculation dysfunction and inflammation implicated in the pathophysiology of LV remodeling are possible links between abnormal LV geometry and adverse cardiovascular events [32]. Second, hypertension plays an essential role in the progression of LV hypertrophy and cardiac-cerebral vascular disease [7, 10, 35]. Third, concentric remodeling is earliest change and a compensatory adaptation to LV systolic pressure overload, but concentric and eccentric hypertrophy have an association with the greater LVMI, which is a marker of more advanced end-organ damage and a leading risk for CVD morbidity and all-cause mortality [9]. In addition, LV remodeling causes LA enlargement, which is a significant predictor of adverse cardiovascular events [36]. Taken together, echocardiographic LV geometry as a key parameter has important implications for enhancing risk stratification and prediction. The early control and reversal of LV remodeling, especially concentric and eccentric hypertrophy may be a therapeutic target for decreasing future adverse events. However, further researches need to explore the mechanisms responsible for cardiac geometry-induced poor outcomes and investigate an effective way to improve prognosis in the general population with LV geometry change.

Based on 8848 hypertensive patients from the Campania Salute Network, de Simone et al. found that LV concentric and eccentric hypertrophy were associated with a marked increase in major cardiovascular risk, whereas no significant risk was detected for LV concentric remodeling [13]. In the Prospective Study of the Vasculature in Uppsala Seniors study, a 2.3-fold increased risk of future CVD was seen for LV concentric hypertrophy as compared to normal group, but LV concentric remodeling and eccentric hypertrophy were not associated with an increased risk of CVD in elderly individuals without myocardial infarction [14]. By assessing all-cause mortality in hypertensive patients, Milani et al. found a significant link between abnormal LV geometry and increased risk of all-cause mortality when compared to normal geometry, and mortality was elevated 2.0-fold in LV concentric remodeling, similar to eccentric hypertrophy [37]. A cohort study in New Zealand reported that LV concentric remodeling and eccentric hypertrophy were independently associated with higher all-cause mortality in people of advanced age [38]. However, our general population-based study found no significant results with respect to the ability of LV concentric remodeling to predict all-cause mortality. A consistent findings were observed in the Losartan Intervention for Endpoint Reduction (LIFE) echocardiography study, Bang et al. demonstrated that LV concentric and eccentric hypertrophy, but not concentric remodeling, were relevant to an increased risk of all-cause mortality in hypertensive patients [15]. A possible explanation for above discrepancy may be attributable to differences in study cohorts, including population selection, sample size, disease type, reported outcomes, follow-up length and ethnicity [12, 35].

Furthermore, current study examined the prognostic relevance of geometric patterns in sex-specific subgroups. After adjusting for the potential confounders, the association between LV eccentric hypertrophy and stroke/CHD incidence was revealed in men, whereas in women, LV concentric hypertrophy was associated with both incident stroke/CHD and all-cause mortality, and eccentric hypertrophy was related to all-cause mortality. These results suggested that echocardiographic predictors of adverse outcomes might differ between men and women, favoring the due consideration of sex-dependent influence in the risk prediction tool. In a prior report, Framingham investigators observed that LV concentric hypertrophy was associated with an increased risk of death and incident CVD in preselected healthy people, particularly in men [17]. In a recent prospective study with 3754 CHD patients, Miller et al. illustrated that LV concentric hypertrophy was correlated with elevated all-cause mortality in both women and men, while eccentric hypertrophy was to increase the risk for all-cause mortality in women but not in men [39]. The reasons for these sex differences in the adaptation of LV remodeling to develop into different outcomes are not clear, but different responses to the same stimuli and altered compensatory pathways in men and women have been demonstrated [10, 39]. In extent, the sex differences may be attributed to sex differences in applicability of the European definitions for LV remodeling on Asian cohorts. Additional pathophysiologic studies are warranted to elucidate the mechanisms underlying these sex-specific associations.

Several limitations exist in our study. First, this was a single center study and some participants were excluded due to a lack of ultrasonic data, which perhaps resulted in a selection bias. Second, we used European definitions on Asian population because of the lack of echocardiographic definitions of LV remodeling profiles for Asians. Third, although we tried to adjust for potential risk factors, there might be residual confounding. In addition, the limited number of end events in this study might restrict the interpretation of results. Therefore, further longitudinal studies involving larger and multiethnic samples with longer follow-up time are warranted to confirm our observation.

## Conclusions

Overall, abnormal echocardiographic LV geometry carried a significant increase in the risk for stroke/CHD and all-cause death incidence. LV eccentric hypertrophy was associated with an elevated risk of incident stroke/CHD in men, whereas concentric or eccentric hypertrophy had an association with the incidence of stroke/CHD or all-cause mortality in women. Thus, the efforts should be made to emphasize the importance of preventing LV remodeling in order to reduce future adverse events in the general population.

## Supplementary Information

**Additional file 1: Supplemental Table 1**. Clinical and echocardiographic characteristics of the included participants.

## Data Availability

The datasets generated and/or analyzed during the current study are available from the corresponding author on reasonable request.

## Abbreviations

LV: Left ventricular
CVD: Cardiovascular disease
MACEs: Major adverse cardiovascular events
BSA: Body surface area
BMI: Body mass index
SBP: Systolic blood pressure
DBP: Diastolic blood pressure
TC: Total cholesterol
TG: Triglyceride
LDL-C: Low-density lipoprotein cholesterol
HDL-C: High-density lipoprotein cholesterol
FPG: Fasting plasma glucose
LAD: Left atrial diameter
IVSd: Interventricular septal thickness
LVIDd: LV end-diastolic internal dimension
LVIDs: LV end-systolic internal dimension
PWTd: Posterior wall thickness
LVEDV: LV end-diastolic volume
LVESV: LV end-systolic volume
LVEF: LV ejection fraction
FS: Fractional shortening
E: Early diastolic peak flow
A: Atrial peak flow
LVM: Left ventricular mass
LVMI: LVM index
RWT: Relative wall thickness
CHD: Coronary heart disease
HRs: Hazard ratios
CIs: Confidence intervals
