# Supplementary material for: Echocardiographic left ventricular geometry profiles for prediction of stroke, coronary heart disease and all-cause mortality in the Chinese community: a rural cohort population study
Source: BMC Cardiovasc Disord. 2021 May 12;21:238. doi: 10.1186/s12872-021-02055-w (PMC8114526; doi:10.1186/s12872-021-02055-w)
Supplement: Supplementary file 1 — Additional file 1: Supplemental Table 1. Clinical and echocardiographic characteristics of the included participants. [file 12872_2021_2055_MOESM1_ESM.docx]

| Supplemental table 1. Clinical and echocardiographic characteristics of the included participants. | | | | |
| --- | --- | --- | --- | --- |
|  | Full cohort (n=9940) | Male (n=4590) | Female (n=5350) | *P* value |
| **Clinical variables** |  |  |  |  |
| Age, years | 53.82 ± 10.48 | 54.30 ± 10.70 | 53.41 ± 10.27 | <0.001 |
| BSA, m^2^ | 1.65 ± 0.18 | 1.74 ± 0.16 | 1.57 ±0.15 | <0.001 |
| BMI, kg/ m2 | 24.82 ± 3.69 | 24.75 ± 3.51 | 24.88 ± 3.84 | 0.10 |
| Smoking, n (%) | 3520 (35.4) | 2646 (57.6) | 874 (16.3) | <0.001 |
| Drinking, n (%) | 2251 (22.6) | 2094 (45.6) | 157 (2.9) | <0.001 |
| Heart rate, bpm | 78.33 ± 1.32 | 76.43 ± 13.09 | 79.95 ± 13.13 | <0.001 |
| History of CVD, n (%) | 798 (8.0) | 328 (7.1) | 470 (8.8) | <0.01 |
| SBP, mmHg | 142.06 ± 23.38 | 143.92 ± 22.48 | 140.46 ± 24.02 | <0.001 |
| DBP, mmHg | 82.12 ± 11.74 | 83.88 ± 11.81 | 80.62 ± 11.47 | <0.001 |
| Hypertension, n (%) | 5000 (50.3) | 2443 (53.2) | 2557 (47.8) | <0.001 |
| Medication for hypertension, n (%) | 1471 (14.8) | 565 (12.3) | 906 (16.9) | <0.001 |
| TC, mmol/L | 5.25 ± 1.09 | 5.19 ± 1.04 | 5.31 ± 1.13 | <0.001 |
| TG, mmol/L | 1.60 ± 1.44 | 1.62 ± 1.61 | 1.57 ± 1.28 | 0.09 |
| LDL-C, mmol/L | 2.95 ± 0.83 | 2.90 ± 0.80 | 3.00 ± 0.86 | <0.001 |
| HDL-C, mmol/L | 1.42 ± 0.39 | 1.42 ± 0.43 | 1.42 ± 0.35 | 0.88 |
| Dyslipidemia, n (%) | 3568 (36.2) | 1676 (36.9) | 1892 (35.6) | 0.18 |
| Estimated GFR, mL/min/1.73 m^2^ | 93.42 ± 15.45 | 94.43 ± 14.59 | 92.56 ± 16.11 | <0.001 |
| FPG, mmol/L | 5.89 ± 1.61 | 5.93 ± 1.63 | 5.86 ± 1.59 | 0.02 |
| Diabetes, n (%) | 1047 (10.5) | 445 (9.7) | 602 (11.3) | 0.01 |
| Medication for diabetes, n (%) | 369 (3.7) | 123 (2.7) | 246 (4.6) | <0.001 |
| **Echocardiographic variables** |  |  |  |  |
| LAD, cm | 3.38 ± 0.40 | 3.47 ± 0.40 | 3.30 ± 0.39 | <0.001 |
| IVSd, cm | 0.87 ± 0.17 | 0.91 ± 0.12 | 0.85 ± 0.11 | <0.001 |
| LVIDd, cm | 4.72 ± 0.42 | 4.91 ± 0.40 | 4.55 ± 0.36 | <0.001 |
| LVIDs, cm | 3.12 ± 0.43 | 3.26 ± 0.43 | 3.01 ± 0.39 | <0.001 |
| PWTd, cm | 0.85 ± 0.10 | 0.88 ± 0.10 | 0.83 ± 0.10 | <0.001 |
| LVM, g | 138.36 ± 36.34 | 154.70 ± 37.08 | 124.34 ± 29.13 | <0.001 |
| LVMI, g/m^2^ | 83.86 ± 19.33 | 89.01 ± 20.09 | 79.43 ± 17.48 | <0.001 |
| RWT | 0.366 ± 0.046 | 0.369 ± 0.047 | 0.37 ± 0.05 | <0.01 |
| LVEDV, ml | 104.41 ± 22.17 | 114.53 ± 22.33 | 95.73 ± 17.95 | <0.001 |
| LVESV, ml | 39.82 ± 13.55 | 44.00 ± 14.50 | 36.24 ± 11.54 | <0.001 |
| SV, ml | 64.58 ± 17.40 | 70.52 ± 18.41 | 59.49 ± 14.68 | <0.001 |
| LVEF, % | 61.80 ± 10.01 | 61.45 ± 10.30 | 62.11 ± 9.75 | <0.01 |
| FS, % | 33.72 ± 7.20 | 33.61 ± 7.45 | 33.82 ± 6.98 | 0.13 |
| E wave, cm/s | 73.20 ± 19.89 | 71.49 ± 18.89 | 74.68 ± 20.60 | <0.001 |
| A wave, cm/s | 76.30 ± 17.83 | 73.68 ± 17.40 | 78.53 ± 17.88 | <0.001 |
| E/A | 1.01 ± 0.38 | 1.02 ± 0.38 | 1.00 ± 0.38 | <0.01 |
| BSA, body surface area; BMI, body mass index; SBP, systolic blood pressure; DBP, diastolic blood pressure; CVD, cardiovascular disease; TC, total cholesterol; TG, triglyceride; LDL-C, low-density lipoprotein cholesterol; HDL-C, high-density lipoprotein cholesterol; GFR, glomerular filtration rate; FPG, fasting plasma glucose; LAD, left atrial diameter; IVSd, interventricular septal thickness; LVIDd, left ventricular end-diastolic internal dimension; LVIDs, left ventricular end-systolic internal dimension; PWTd, posterior wall thickness; LVM, left ventricular mass; LVMI, left ventricular mass index; RWT, relative wall thickness; LVEDV, left ventricular end-diastolic volume; LVESV, left ventricular end-systolic volume; SV, systolic volume; LVEF, left ventricular ejection fraction; FS, fractional shortening; E, early diastolic peak flow; A, atrial peak flow. | | | | |
